# Electronic Structure and Spectroscopy of CuBe

**DOI:** 10.1021/acs.jpca.4c08565

**Published:** 2025-02-13

**Authors:** Arianna Rodriguez, Sophia Vadachkoria, Francesco A. Evangelista, Michael C. Heaven

**Affiliations:** Department of Chemistry, Emory University, Atlanta, Georgia 30322, United States

## Abstract

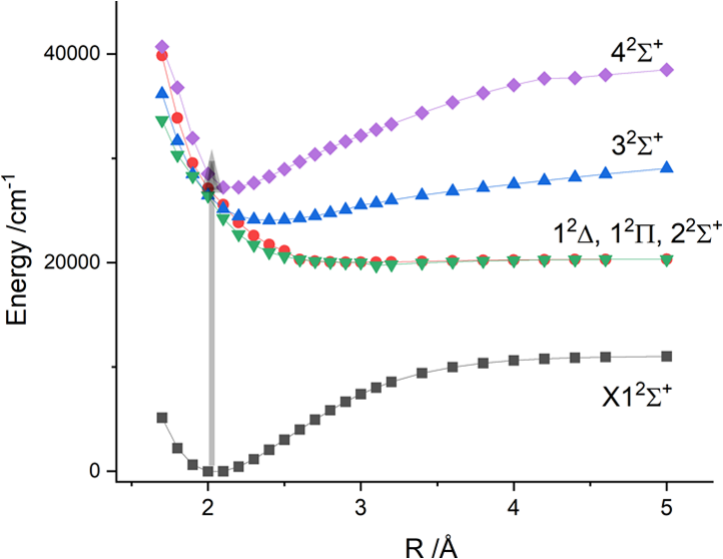

Electronic spectra for CuBe were recorded using resonantly enhanced one-color, two-photon ionization techniques. Several vibronic progressions were observed, permitting characterization of the 4^2^Σ^+^, 5^2^Σ^+^, 3^2^Π and 4^2^Π excited states. Rotational resolution was achieved for a few bands, permitting determination of the ground state rotational constant. Attempts to record laser-induced fluorescence or two-color ionization spectra were unsuccessful. This was attributed to predissociation of the excited states. Density functional theory and *ab initio* electronic structure calculations were used to facilitate assignment of the observed states. Potential energy curves obtained from these calculations indicated that states arising from the Cu(3d^9^4s^2^) + Be(2s^2^) dissociation asymptote were well-positioned to cause predissociation of the observed states.

## Introduction

Diatomic molecules consisting of an alkali metal (Alk = Li, Na, K, Rb, Cs, Fr) bound to an alkaline earth metal (AE = Be, Mg, Ca, Sr, Ba, Ra) have been investigated in recent years due to their potential suitability for experiments that involve ultracold molecules.^1−15^ In part, this interest stems from the fact that Alk-AE diatomics may be manipulated using both electric and magnetic fields. Ultracold Alk-AE diatoms may be produced by photoassociation of ultracold atoms or direct laser cooling.^1^ The latter requires the existence of an electronic transition that has an origin band (vibrational 0 ↔ 0) that is strongly favored by the Franck–Condon factor (e.g., FCF > 0.9). The Alk-AE dimers have their outermost electron in the Alk-centered ns orbital, which is nominally nonbonding. Strongly allowed transitions are associated with the ns → np transition. To a first approximation the np orbital is also nonbonding, so the transitions may be expected to have near diagonal FCF distributions.

A summary of both experimental and theoretical studies of Alk-AE diatoms has been provided by Ladjimi et al.^1^ Recent experimental studies of LiBe^4^ and LiMg^5^ were carried out by the Heaven group at Emory. For LiMg, laser-induced fluorescence (LIF) and dispersed laser-induced fluorescence (DLIF) techniques were used to characterize the X1^2^Σ^+^, 1^2^Π and 2^2^Σ^+^ states.^5^ The results confirmed the predictions from high-level computational models. A similar study of LiBe^4^ yielded rotationally resolved data for the 2^2^Σ^+^–X1^2^Σ^+^ transition and validated high-level computational predictions that this transition is promising for direct laser cooling.^2^

Molecules consisting of an alkali metal bound to a coinage metal (CM = Cu, Ag, Au) are isoelectronic with the Alk-AE dimers.^16−19^ The Au-AE diatoms are the most studied examples of this family. Mass spectrometric measurements have been used to determine the bond energies, which range from 14,810 cm^–1^ for AuMg to 22,910 cm^–1^ for AuBa.^17−19^ Gas phase electronic spectra have been reported for Au-AE species with AE = Be, Mg, Ca, Sr and Ba.^20−24^ The ground states are X1^2^Σ^+^, arising from the Au(5d^10^6s) + AE(ns^2^) dissociation asymptotes. The lower energy electronic transitions for AuBe and AuMg are associated with the Au- centered 5d^10^6s → 5d^9^6s^2^ excitation. For the heavier AE elements, the lower energy transitions are centered on the AE atom. Spectroscopic data are not available for the analogous Cu-AE and Ag-AE diatoms.

In the present work we have examined electronic transitions of CuBe. The X1^2^Σ^+^ ground state has been the subject of a few previous computational studies,^16,19,25^ which predicted bond dissociation energies in the range of 6210 to 11,350 cm^–1^. Theoretical predictions for the electronically excited states have not been published. Here we present electronic spectra for CuBe recorded using the resonantly enhanced two-photon ionization (RE2PI) technique. To facilitate interpretation of these data, the results of *ab initio* and density functional theory (DFT) calculations for excited state potential energy curves are also reported.

## Methods

Gas phase CuBe was generated using pulsed laser ablation and supersonic expansion techniques.^4^ Initially we attempted to generate CuBe by ablating a rod of copper–beryllium alloy, but the concentration of Be (2% by weight) was too low for CuBe to compete with the formation of Cu_2_. Consequently, we switched to a target consisting of a beryllium rod coated with powdered copper. Continuous rotation and translation of the rod was used to avoid burning holes in the coating and pitting of the Be rod. The rod was mounted in a copper block, so that the translation and rotation also provided some transfer of copper by abrasion. Typically, the rod needed to be recoated with Cu power after a week of experimental work.

The fundamental output from a Nd/YAG laser (1064 nm, Continuum Minilite) was used for ablation. The resulting metal vapor was entrained in a flow of He carrier gas that was provided by a pulsed valve (Parker–Hannifin series 9) operating with a backing pressure of 5 atm. The gas was supersonically expanded into vacuum through the 2 mm diameter orifice of the rod mount. The expanding gas was sampled by a conical skimmer into a differentially pumped analysis chamber. This housed a Wiley–McLaren time-of-flight mass spectrometer that was used to analyze the ablation products and provide mass-selected ion detection.

Spectroscopic measurements were carried out using the RE2PI technique. These were one-color two-photon excitations, employing excitation laser photon energies that exceeded half of the CuBe ionization energy. The laser used for this study was a Continuum ND6000 tunable dye laser pumped by Powerlite 8000 Nd/YAG laser. Light from the ND6000 was frequency doubled to generate near ultraviolet (UV) wavelengths (280–330 nm). The dye laser fundamental had a line width of approximately 0.3 cm^–1^ fwhm. On frequency doubling the line width increased to 0.5 cm^–1^.

A few experiments used the overlapped beams from two pulsed lasers to search for two-color RE2PI signals.

## Results and Analyses

Preliminary DFT calculations predicted an ionization energy (IE) for CuBe of 56,070 cm^–1^. Consequently, we began our search for one-color RE2PI signals at wavelengths corresponding to 30,000 cm^–1^. Cu has two stable isotopes with significant natural abundances (69% ^63^Cu and 31% ^65^Cu), while the only stable isotope of beryllium is ^9^Be. Consequently, the signals from CuBe appeared as an easily recognized doublet in the mass spectrum. RE2PI spectra for CuBe were recorded using simultaneous, independent monitoring of the mass spectrometer signals for the two isotopologues.

Descriptions of the observed spectra are facilitated by a brief consideration of the molecular states that arise from the low energy atomic states. Table 1 lists the atomic state dissociation asymptotes relevant to this study and the correlated molecular states (these energies have been adjusted to spin-free values as the calculations that they will be compared with did not include the spin–orbit interactions). The numberings of the molecular states correspond to the ascending energy ordering for the states of a given symmetry. Note that the ordering used here is for nearly separated atoms. Some of these states change order at short internuclear distances, but the numbering of Table 1 is retained. State assignments used in the following descriptions of spectra are justified in subsequent sections.

**Table 1:** Correlation of Molecular Electronic States of CuBe with Separated Atom Limits^a^

Be	Cu	energy^26^ (cm^–1^)	states Λ = 0	states Λ = 1	states Λ = 2	states Λ = 3
2s^2^, ^1^S_g_	4s, ^2^S_g_	0	1^2^Σ^+^			
2s^2^, ^1^S_g_	3d^9^4s^2^, ^2^D_g_	12,019.8	2^2^Σ^+^	1^2^Π	1^2^Δ	
2s2p, ^3^P_u_	4s, ^2^S_g_	21,980.2	3^2^Σ^+^	2^2^Π		
2s^2^, ^1^S_g_	4p, ^2^P_u_	30,700.9	4^2^Σ^+^	3^2^Π		
2s2p, ^3^P_u_	3d^9^4s^2^, ^2^D_g_	33,999.9	5^2^Σ^+^	4^2^Π	2^2^Δ	1^2^Φ
			6^2^Σ^+^	5^2^Π	3^2^Δ	
			1^2^Σ^–^	6^2^Π		

a This table omits the states of quartet spin multiplicity.

**Figure 1:**
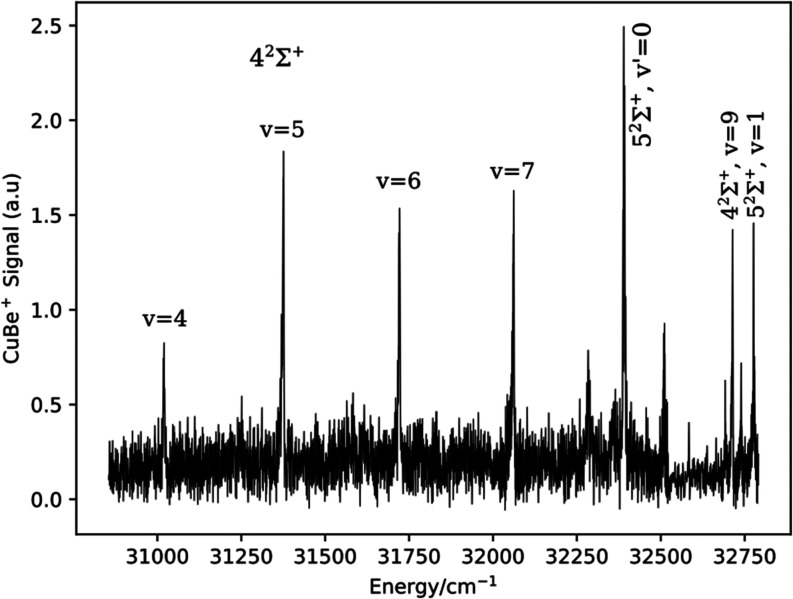
Survey spectrum for CuBe, 29,950–32,770 cm^–1^.

The lowest energy features observed in the spectrum defined a vibrational progression that could be followed from 31018 to 32,720 cm^–1^, as shown in Figure 1. These bands exhibited red-shaded rotational contours that did not resolve individual rotational lines. However, the band contours were consistent with the 4^2^Σ^+^–X1^2^Σ^+^ transition. The ^63^CuBe/^65^CuBe isotope shifts were large enough to show that the first band observed in this progression was not the origin band. The positions of the band intensity maxima for ^63^CuBe are listed in Table 2. The band positions and isotope shifts for ^65^CuBe are presented in the Supporting Information.

Isotope shifts were used to estimate the vibrational numbering of the 4^2^Σ^+^–X1^2^Σ^+^ progression. The first step in this process was to fit the band maxima positions for ^63^CuBe to the expression
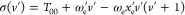
1where *T*_00_ is the energy of the transition origin, ω_e_^′^ and ω_e_*x*_e_^′^ are the upper state Morse vibrational constants and v′ is the vibrational quantum number. The isotope shifts were calculated from the equation
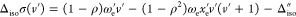
2where ρ is the reduced mass ratio  and Δ_iso_^″^ is the isotopic shift of the ground state zero-point level. Using the vibrational constants from our *ab initio* potential energy curve for the ground state (see below) we obtained an estimate for Δ_iso_^″^ of 0.47 cm^–1^. The vibrational numbering that gave the best fit to the isotope shifts assigns the band at 31,018 cm^–1^ to v′ = 4. With this choice we obtained *T*_00_ = 29,523(16), ω_e_^′^ = 393.3(48) and ω_e_*x*_e_^′^ = 3.87(34) cm^–1^ for the ^63^CuBe 4^2^Σ^+^ state.

**Figure 2:**
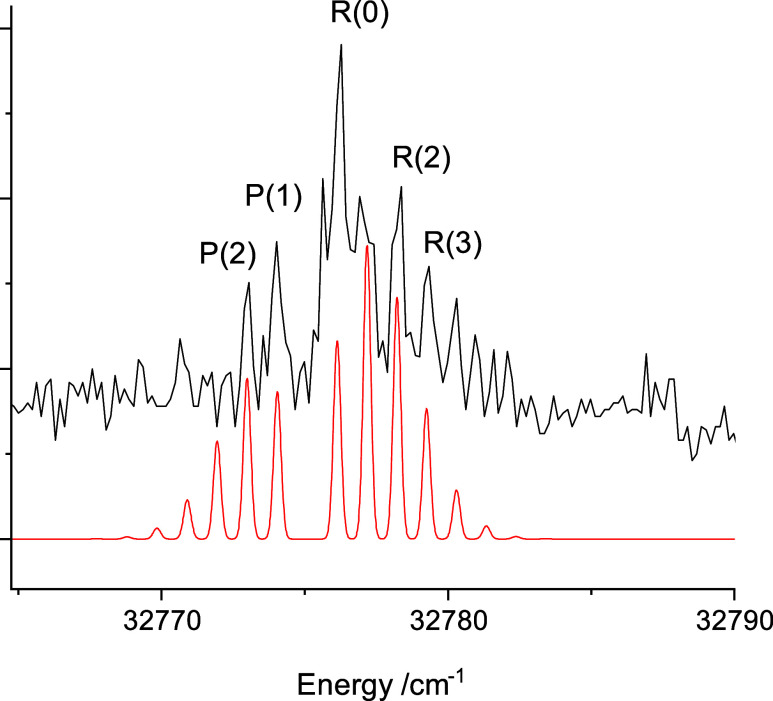
Rotational structure of the 5^2^Σ^+^–X1^2^Σ^+^ 1–0 band.

Transitions to the 5^2^Σ^+^ v′ = 0 and 1 levels were observed at 32,391.5 and 32,776.8 cm^–1^. The rotational structures of these bands were resolved as the upper and lower state rotational constants were very similar. Figure 2 shows the rotational structure of the v′ = 1 band, along with a simulation generated using the PGOPHER software package.^27^ The difference between the upper and lower state rotational constants (*B*′ = 0.526(5) and *B*″ = 0.522(8) cm^–1^) was statistically insignificant. Modeling indicated a rotational temperature of approximately 4 K, so the small number of observable levels limited the accuracy with which the rotational constants could be determined. The isotope shift for the v′ = 0 level was below the resolution of our measurements. For v′ = 1 the shift was 0.68(12) cm^–1^, consistent with the value expected based on the Δ*G*_1/2_′ value. The similarity of the upper and lower state rotational constants and the fact that we could not observe the v′ > 1 levels suggested a near diagonal FCF distribution. Lastly, we note that the energy of the 4^2^Σ^+^ v′ = 8 level is missing from Table 1 because this weak band was obscured by the more intense 5^2^Σ^+^ v′ = 0 feature.

**Figure 3:**
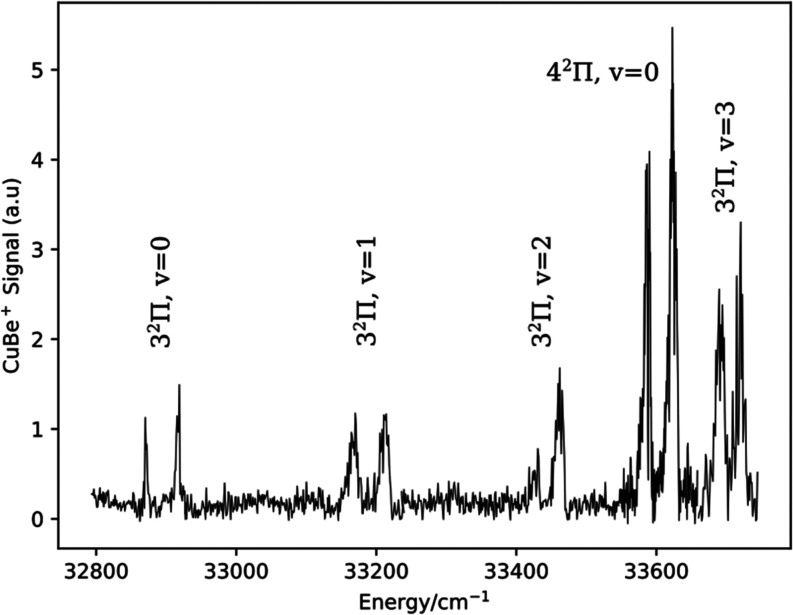
Survey spectrum for CuBe, 32,820–33,950 cm^–1^.

Beginning at 32,871.0 cm^–1^ we encountered vibronic features that displayed the characteristic spin–orbit split components of ^2^Π states. The positions of the band intensity maxima for the assigned members these progressions are listed in Table 2. Figure 3 is a low-resolution survey scan that shows a vibrational progression for the 3^2^Π–X1^2^Σ^+^ transition and the origin band of the 4^2^Π–X1^2^Σ^+^ transition. Rotational band contours for both the 3^2^Π and 4^2^Π states were consistent with regular spin–orbit splitting [E(^2^Π_3/2_) > E(^2^Π_1/2_)], but the resolution was not sufficient to reach a firm conclusion on this point. The vibrational intervals between the 3^2^Π–X1^2^Σ^+^ bands were erratic but could be followed reliably up to v′ = 3. Fitting to the Morse energy expression yielded molecular constants with relatively large error ranges (^63^CuBe; *T*_00_ = 32723(9), ω_e_ = 308(19), ω_e_x_e_ = 9.0(47), standard deviation 5.5 cm^–1^ for 3^2^Π_1/2_ and *T*_00_ = 32922(12), ω_e_ = 302(25), ω_e_*x*_e_ = 9.1(60), standard deviation 6.9 cm^–1^ for 3^2^Π_3/2_).

**Table 2:** Band Maxima for Assigned Transitions of ^63^CuBe

state	Ω	vibrational level	energy^a^ /cm^–1^
4^2^∑^+^	1/2	4	31,018.2
4^2^∑^+^	1/2	5	31,373.1
4^2^∑^+^	1/2	6	31,715.4
4^2^∑^+^	1/2	7	32,058.4
4^2^∑^+^	1/2	9	32,712.3
5^2^∑^+^	1/2	0	32,389.4
5^2^∑^+^	1/2	1	32,775.1
3^2^Π	1/2	0	32,871.1
3^2^Π	3/2	0	32,916.7
3^2^Π	1/2	1	33,167.6
3^2^Π	3/2	1	33,211.7
3^2^Π	1/2	2	33,431.0
3^2^Π	3/2	2	33,461.1
3^2^Π	1/2	3	33,690.6
3^2^Π	3/2	3	33,719.4
4^2^Π	1/2	0	33,586.3
4^2^Π	3/2	0	33,623.2
4^2^Π	1/2	1	33,997.7
4^2^Π	3/2	1	34,040.9
4^2^Π	1/2	2	34,406.7
4^2^Π	3/2	2	34,456.5
4^2^Π	1/2	3	34,802.1
4^2^Π	3/2	3	34,843.5
4^2^Π	1/2	4	35,176.0
4^2^Π	3/2	4	35,211.9

a 1-σ errors, ±1.0 cm^–1^

**Figure 4:**
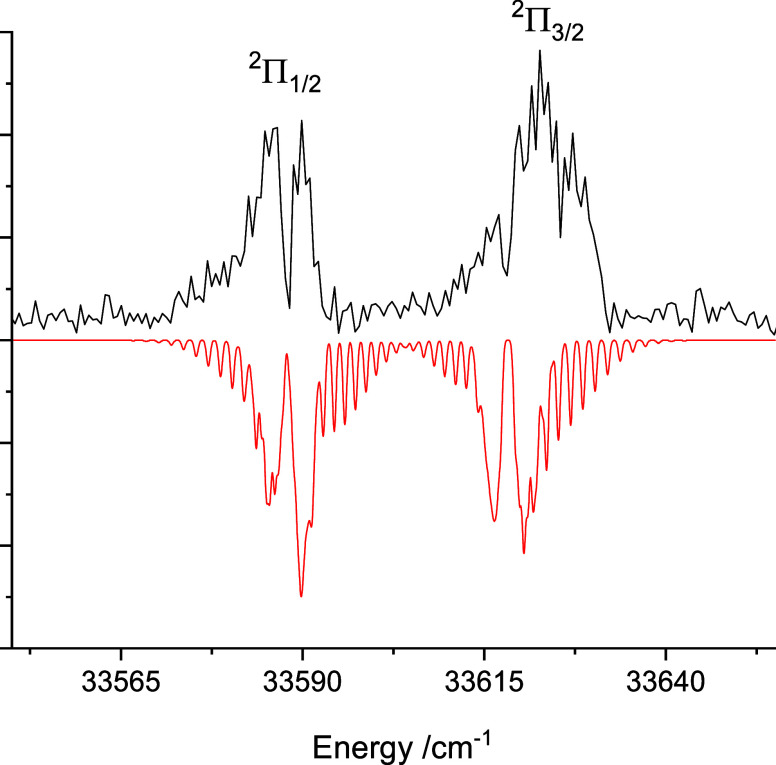
Rotational structure of the 4^2^Π–X1^2^Σ^+^ 1–0 band.

**Figure 5:**
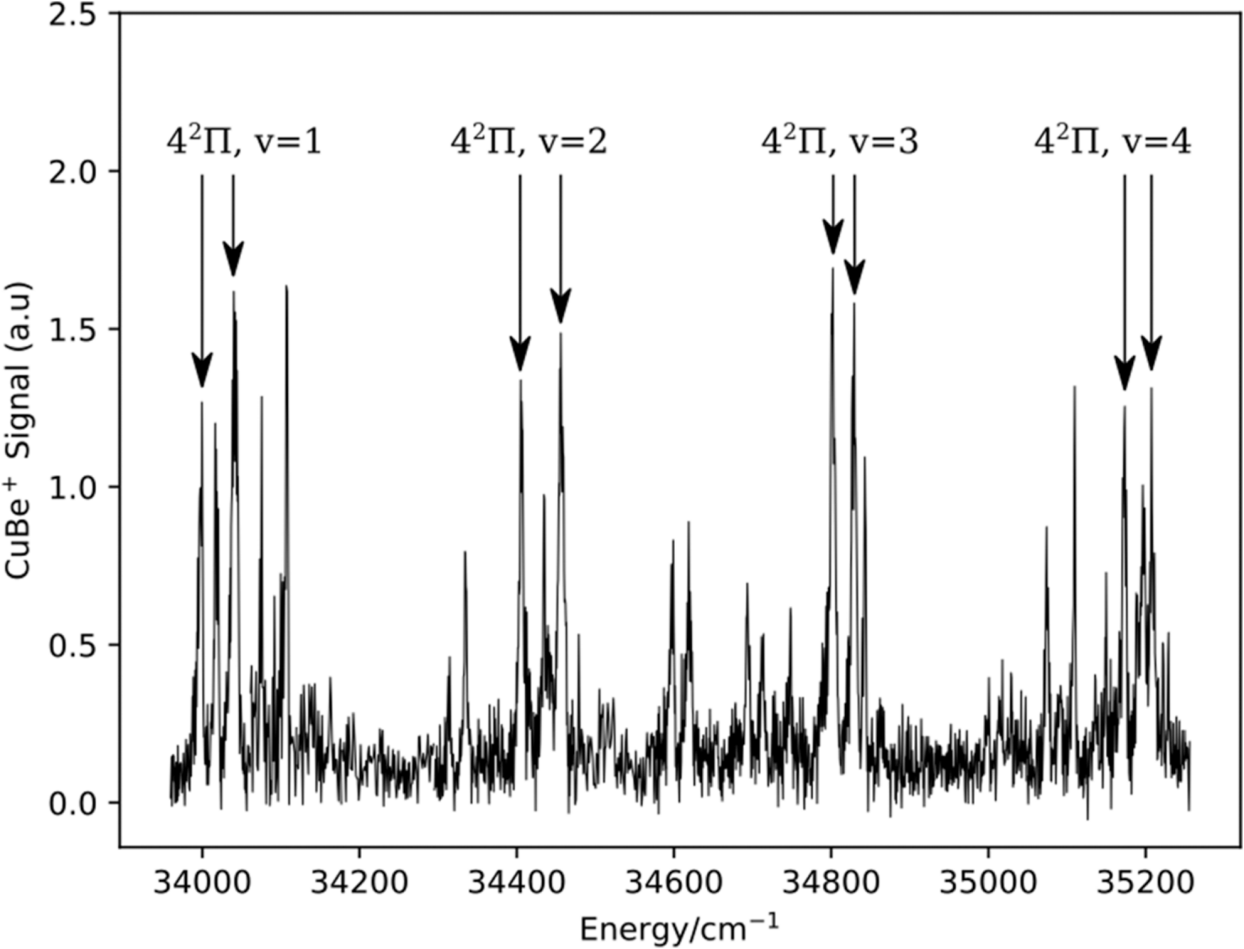
Survey spectrum for CuBe, 33,920–35,250 cm^–1^.

The 4^2^Π–X1^2^Σ^+^ 0–0 band was the most intense feature within the spectral range examined. A partially resolved rotational contour for this band is shown in Figure 4, along with an approximate PGOPHER simulation. For this model the upper state rotational constant was B′ = 0.520 cm^–1^, the spin–orbit splitting constant *A*′_SO_ = 31.5 cm^–1^ and the rotational temperature was 15 K. The ground state rotational constant was held at the value obtained by fitting the 5^2^Σ^+^–X1^2^Σ^+^ 1–0 band. The vibrational progression of the 4^2^Π–X1^2^Σ^+^ transition was followed up to the v′ = 4 level. Figure 5 shows these bands, and it also reveals the rapidly increasing congestion and complexity of the spectrum as the energy is increased. As for the 3^2^Π–X1^2^Σ^+^ progression, fitting of the 4^2^Π–X1^2^Σ^+^ band centers to the Morse energy level expression yielded molecular constants with substantial uncertainties. For ^63^CuBe 4^2^Π the constants were *T*_00_ = 33586.5(41), ω_e_ = 428.2(60), ω_e_*x*_e_ = 6.1(11), standard deviation 3.1 cm^–1^ for Ω = 1/2 and *T*_00_ = 33621.6(44), ω_e_ = 443.1(44), ω_e_*x*_e_ = 9.0(12), standard deviation 3.2 cm^–1^ for Ω = 3/2. We have not attempted to assign the less intense features in Figure 5, but it is evident that bands from other ^2^Σ^+^ and ^2^Π electronic states are present in this range. The energies of these unassigned bands are listed in the Supporting Information.

In addition to the one-color RE2PI measurements we also attempted to observe CuBe using two-color RE2PI and LIF. The 355 nm light from a frequency-tripled Nd/YAG laser was used for the ionizing photon, but no two-color contributions were observed in the flux of CuBe^+^ ions. The LIF measurements detected only scattered laser light.

### Electronic Structure Calculations

Previous calculations for the ground state of CuBe have yielded a wide range of values for the dissociation energy. We have revisited this problem, in part to help with the selection of methods that would be suitable for calculations of the excited state properties. A collection of past and present results is provided in Table 3.

**Table 3:** Calculated Molecular Constants for ^63^CuBe

method	basis	ω_e_/cm^–1^	*R*_e_/Å	*D*_e_/cm^–1^
SDCI^28^	[9s 7p 4d 3f 1g/6s 3p 3d 1f]	449	2.128	6210
CPF^28^	[9s 7p 4d 3f 1g/6s 3p 3d 1f]	435	2.116	7420
DFT (TPSSh)^19^	def2-QZVPP	465	2.080	11350
RCCSD(T)^16^	aug-cc-pWCV5Z + ECPs + bond functions	506	2.072	9108
RCCSD^a^	cc-pwCVQZ	424	2.120	6082
RCCSD(T)^a^	cc-pwCVQZ	457	2.075	8150
RCCSD(T) + DKH2^a^	cc-pwCVQZ-DK	480	2.036	9327
DFT (B3LYP) + DKH2^a^	cc-pwCVQZ-DK	487	2.046	11155

a This work.

One of the earliest calculations was carried by Bauschlicher et al.^28^ They used the singles and doubles configuration interaction (SDCI) and coupled pair functional (CPF) methods with a basis set of quadruple-ζ (QZ) quality. The CPF results were considered to be the most accurate.

Restricted coupled cluster calculations^29^ show that the bond dissociation energy is significantly influenced by the inclusion of triple excitations. Smiałkowski and Tomza^16^ carried out restricted coupled cluster with singles, doubles, and perturbative triples [RCCSD(T)] calculations with basis sets of 5Z quality, obtaining higher bond energies than Bauschlicher et al.^28^ In the present work, we ran both RCCSD and RCCSD(T) calculations using the Psi4 electronic structure package^29^ with the cc-pwCVQZ basis sets.^30,31^ Our most accurate predictions correlate the 1s shell of Be and 3s and 3p shells of Cu in addition to the atomic valence shells, and account for scalar relativistic corrections treated at the second-order Douglas–Kroll–Hess level (DKH2).^32^ Our computations show a 2068 cm^–1^ increase in the dissociation energy when the (*T*) correction is added on top of RCCSD, and an additional contribution of 1176 cm^–1^ due to scalar relativistic effects. Our best estimate of the bond dissociation energy (9327 cm^–1^) is 219 cm^–1^ larger than the value reported by Smiałkowski and Tomza.^16^

Calculations for the ground and excited states were performed using the time-dependent-density functional theory (TD-DFT) method with the inclusion of DKH2, as implemented by the Orca 6.0.1 software package.^33^ The B3LYP functionals and aug-cc-pVQZ-DK basis set were used to predict properties of the ground state and the first 10 excited states. At this level of theory, the ground state bond dissociation energy was 11,155 cm^–1^, 22% above our RCCSD(T) result. Potential energy curves were constructed from single-point energies using a grid spaced by 0.1 Å over the internuclear separation range from 1.6 to 4.5 Å. An additional calculation was made at 5 Å to approximate the dissociation limit.

**Figure 6:**
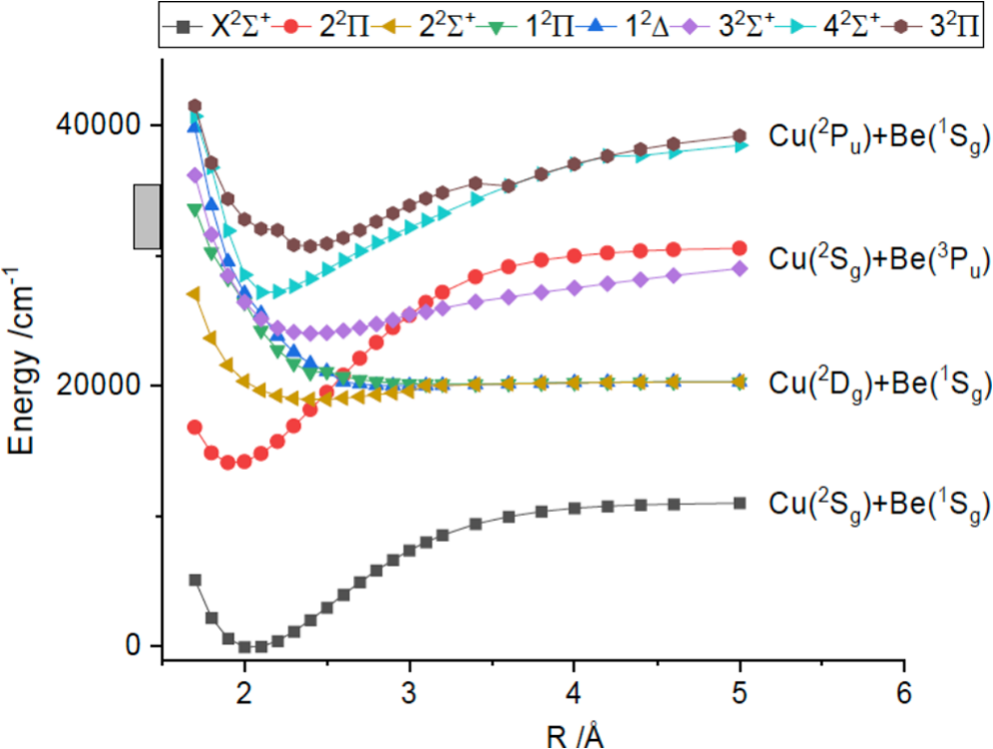
Potential energy curves for CuBe from TD-DFT calculations. The gray bar on the vertical axis shows the energy range probed by means of laser excitation.

TD-DFT potential energy curves for the excited states are shown in Figure 6. In this plot, it can be seen that the first excited state at the ground state equilibrium distance is 2^2^Π, which correlates with the Be(2s2p, ^3^P_u_) + Cu(3d^10^4s, ^2^S_g_) dissociation asymptote. It is the most deeply bound state in this energy range. The oscillator strength for 2^2^Π–X1^2^Σ^+^ was predicted to be small (*f*_osc_ = 8 × 10^–4^) as it is associated with the spin-forbidden Be (2s2p, ^3^P_u_)–(2s^2^, ^1^S_g_) transition. The 1^2^Π and 1^2^Δstates derived from the Be(2s^2^, ^1^S_g_) + Cu(3d^9^4s^2^, ^2^D_g_) dissociation limit were entirely repulsive, while the 2^2^Σ^+^ state was mostly repulsive, but had a shallow minimum near *R* = 2.4 Å. At shorter internuclear distances (<2.4 Å) the repulsive limbs of these states interact with several higher energy states. The limitations of the TD-DFT calculations were indicated by the atomic energy intervals defined by the dissociation asymptotes. The energies of the first three excited state limits were predicted to be 9308, 19,556, and 27,482 cm^–1^, as compared to the experimentally determined values of 12,019.8, 21,980.2, and 30,700.9 cm^–1^.

EE-EOM-CCSD calculations,^34^ implemented using the Orca 6.0.1 software package,^33^ were explored as a potentially more reliable method for the treatment of the excited states. The cc-pwCVTZ-DK basis sets were used, and scalar relativistic effects were included using the DKH2 Hamiltonian. The ground state potential energy curve derived from this model was more deeply bound than the RCCSD potential, but it did not recover the full effect of the neglected triple excitations. The potential energy curves for 10 excited states were generated and the results (shown in Figure S1 of the Supporting Information) were qualitatively similar to those of the TD-DFT calculations. The energies of the first three atomic excited state asymptotes were 9543, 21,958, and 29,120 cm^–1^. EE-EOM-CCSD also predicted that the first excited state was the deeply bound 2^2^Π state, with molecular constants of *T*_e_ = 12,408, *D*_e_ = 16,600, ω_e_ = 518 cm^–1^ and *R*_e_ = 1.91 Å (where the dissociation limit correlates with Cu(^2^S_g_) + Be(^3^P_u_)). The corresponding constants derived from the TD-DFT calculations were *T*_e_ = 14,067, *D*_e_ = 16,500 (relative to Cu(^2^S_g_) + Be(^3^P_u_)), ω_e_ = 544 cm^–1^ and *R*_e_ = 1.94 Å. Several of the EE-EOM-CCSD potential energy curves exhibited discontinuities and root-flipping problems at energies above 20,000 cm^–1^ (evident in Figure S1). Neither the TD-DFT or EE-EOM-CCSD were able to provide reliable results when the states arising from the Cu(^2^D_g_) + Be(^3^P_u_) asymptote were included. Consequently, we present the TD-DFT results in Figure 6 as the EE-EOM-CCSD calculations were less stable.

The IE for CuBe was determined using DFT and IP-EOM-CCSD calculations. Adiabatic energies of 56,070 and 52,251.4 cm^–1^ were obtained.

## Discussion

Electronic structure calculations indicate that predissociation may be responsible for the lack of LIF or two-color ionization signals from CuBe. In Figure 6 the repulsive potentials from the Be(2s^2^, ^1^S_g_) + Cu(3d^9^4s^2^, ^2^D_g_) and Be(2s2p, ^3^P_u_) + Cu(3d^10^4s, ^2^S_g_) dissociation limits are well-positioned to destabilize higher energy states in the 25,000–40,000 cm^–1^ range. It appears that the predissociation rates were much greater than the radiative decay rates, but not high enough to prevent partial rotational resolution for a small subset of bands. Resonantly enhanced two-color photoionization was not observed due to the limitations of pulse timing and the imperfect spatial overlap of the uncorrelated transverse mode structures of the two lasers (problems that are not present when a single beam is used for one-color two-photon ionization). Being limited to one-color ionization we could not achieve a direct measurement of the IE, but note that the detection of a band at 31018 cm^–1^ establishes an upper bound of IE < 62,150 cm^–1^ (including a correction for the local electric field of the mass spectrometer).

The rotational temperatures obtained by modeling the band contours may also provide some insight concerning the predissociation mechanism. Heterogeneous predissociation (ΔΩ = ±1) is mediated by rotational motion, resulting in a nonradiative decay component with a rate that is proportional to *J*(*J* + 1). Hence, the variability of the rotational temperatures obtained by contour modeling may be an artifact of rotationally dependent predissociation.

As the states of CuBe examined did not fluoresce the only information obtained for the ground state was the rotational constant for the v″ = 0 level. The value obtained, *B*_0_ = 0.522(8) cm^–1^ corresponds to an average internuclear separation of *R*_0_ = 2.03(3) Å, consistent with the range of values presented in Table 3.

Assignments for the electronically excited states were based partly on extrapolations of the vibrational manifolds to the atomic state dissociation limits. In this analysis we adopted the RCCSD(T) value of *D*_0_″ = 9087 cm^–1^ for the ground state dissociation energy, but the following arguments can accommodate large uncertainties in this value. As described above, the vibrational numbering for lowest energy vibrational progression observed in this study was established using the ^63^Cu/^65^Cu isotope shifts. A Morse extrapolation of the progression to the dissociation limit (*D*_0_ = 9795 cm^–1^) yielded an energy (relative to X1^2^Σ^+^, v″ = 0) of 39320 cm^–1^. Subtraction of the ground state D_0_″ energy places the estimated dissociation limit roughly 30,233 cm^–1^ above the separated ground state atoms, consistent with the 4^2^Σ^+^ state derived from the Cu(3d^10^4p, ^2^P_u_) + Be(2s^2^, ^2^S_g_) configuration.

The second excited state observed was also of ^2^Σ^+^ symmetry, displaying just the v′ = 0 and 1 vibrational levels separated by Δ*G*_1/2_ = 387 cm^–1^. Assignment of this state to the closest reasonable dissociation limit (Cu(3d^9^4s^2^, ^2^D_g_) + Be(2s2p, ^3^P_u_), 5^2^Σ^+^) would indicate a bond dissociation energy of about 10700 cm^–1^.

The first band with the characteristics of a ^2^Π–X1^2^Σ^+^ transition was observed near 32,871 cm^–1^, with a ^63^Cu/^65^Cu isotope shift that was below the resolution of our measurements. Hence, this feature was assigned as the 0–0 band. The energy of the upper state indicates that it is 3^2^Π, derived from the Cu(3d^10^4p, ^2^P_u_) + Be(2s^2^, ^1^S_g_) configuration. Combining the transition energy with the RCCSD(T) estimate for the ground state dissociation energy yielded a dissociation energy for the 3^2^Π state of *D*_0_′ = 6920 cm^–1^. We note that both the dissociation energy and the harmonic vibrational frequency for the 3^2^Π state are smaller than those of the 4^2^Σ^+^ and 5^2^Σ^+^ states. The second ^2^Π state observed in this study can be reasonably assigned as 4^2^Π derived from the Cu(3d^9^4s^2^, ^2^D_g_) + Be(2s2p, ^3^P_u_) configuration. The bond dissociation energy resulting from this choice is *D*_0_′ = 9500 cm^–1^.

The magnitude of the spin–orbit coupling constants for both the 3^2^Π and 4^2^Π states indicate substantial mixing of the electronic states at energies above 30,000 cm^–1^. The simplest case to analyze in this context is the spin–orbit coupling constant of the 3^2^Π state. If state mixing were minimal, Van Vlecks’ pure precession approximation^35,36^ would predict that the spin–orbit coupling constant of the Cu(3d^10^4p, ^2^P) atomic state (ζ_4*p*_ = 166 cm^–1^)^26^ would be approximately conserved in the molecule. Clearly the observed spin–orbit splittings, ranging from 33 to 38 cm^–1^ are not consistent with the isolated states model. Similarly, the 4^2^Π spin–orbit splittings of 32 and 41 cm^–1^ do not reflect the atomic Cu(3d^9^4s^2^, ^2^D) spin–orbit constant,^26^ ζ_3*d*_ = 817 cm^–1^. Given the tangle of potential energy curves revealed by the electronic structure calculations (cf. Figure 6) it is not surprising that the electronic states are mixed and the vibronic energy levels deviate from traditional polynomial models.

Table 4 presents calculated and observed molecular constants for the 4^2^Σ^+^ and 3^2^Π states. Both the TD-DFT and EE-EOM calculations underestimated the excitation energies, with the largest errors associated with the latter. EE-EOM appeared to give slightly better predictions for the dissociation energies, but the experimental estimates depend on the value for the ground state dissociation energy provided by the RCCSD(T)-DKH2 calculations. The calculated ω_e_ and *R*_e_ values for the 4^2^Σ^+^ and 3^2^Π states were obtained by fitting the Morse potential energy function to the single-point energies surrounding the equilibrium distance (typically the range from 1.8 to 3.0 Å). As can be seen in Figure S1, the EE-EOM potential energy curve for 4^2^Σ^+^ exhibited a discontinuity at *R* = 2.1 Å. Prior to fitting this curve, the data for *R* ≤ 2.1 Å were shifted upward by 1000 cm^–1^. The resulting ω_e_ value was in reasonably good agreement with the experimental value. The 4^2^Σ^+^ potential energy curve from the TD-DFT calculations was not well-represented by the Morse function. The repulsive limb below 2.1 Å rose too sharply, producing a high value for the harmonic vibrational constant. Overall, we consider that the present calculations were sufficient to provide guidance for interpretation of the observed states, but not adequate for quantitative predictions at energies above 25,000 cm^–1^.

**Table 4:** Observed and Calculated Molecular Constants for the 4^2^Σ^+^ and 3^2^Π States of CuBe

	*T*_0_	*D*_0_	ω_e_	*R*_e_	*B*_e_
4^2^Σ^+^, exp	29,523	10,256	393	x	x
4^2^Σ^+^, TD-DFT	27,152	11,104	554	2.16	0.458
4^2^Σ^+^, EE-EOM	25,896	9527	372	2.14	0.461
3^2^Π, exp	32,894	6894	308	x	x
3^2^Π, TD-DFT	30,735	8248	321	2.38	0.380
3^2^Π, EE-EOM	28,996	6933	338	2.36	0.383

One of the motivations for the present study was to see if CuBe may be a suitable molecule for laser cooling. The answer is most probably not. The states that we were able to characterize all appeared to be predissociated. The 2^2^Π state predicted by the electronic structure calculations should be stable as there are no repulsive potential energy curves passing near the bottom of the state. However, there are two problems for any attempt to laser cool via the 2^2^Π–X1^2^Σ^+^ transition. First, the equilibrium distance for 2^2^Π is about 0.1 Å shorter than that of the ground state. A calculation of the FCF’s yields values of 0.512, 0.367, and 0.104 for the 0–0, 1–0 and 2–0 bands. The second problem is the low oscillator strength of the 2^2^Π–X1^2^Σ^+^ transition.

Despite the poor outlook for direct laser cooling, CuBe may still be of interest for studies conducted at ultracold temperatures. Useful ground state properties include a permanent electric dipole moment (estimated to be 0.8 D^16^), the magnetic moment of the Cu 4s electron and the possibility of using the predissociated excited states for rotationally resolved state read-out.

## Conclusions

Electronic transitions of CuBe have been investigated in the gas phase using the technique of resonantly enhanced two-photon ionization. Gas jet expansion was used to cool the molecules to rotational temperatures in the 5–15 K range. Electronic transitions above 30,000 cm^–1^ were accessible using these techniques. Vibrational progressions were observed for the 4^2^Σ^+^, 5^2^Σ^+^, 3^2^Π and 4^2^Π states. A small subset of these bands exhibited resolved rotational structure, permitting determination of the upper and lower state rotational constants. One-color photoionization measurements established an upper bound of 62,150 cm^–1^ for the ionization energy. Attempts to observe LIF or two-color photoionization were unsuccessful, most probably due to predissociation. Extensive mixing of the excited states was indicated by the low values of the ^2^Π state spin–orbit coupling constants.

Electronic structure calculations were used to guide the interpretation of the experimental data. Ground state potential energy curves were generated using the DFT, RCCSD, and RCCSD(T) methods. The latter (including scalar relativistic effects) yielded a bond dissociation energy of *D*_0_″ = 9087 cm^–1^ and a *B*_0_″ rotational constant that agreed with the measured value. Excited state potential energy curves were generated using the TD-DFT and EE-EOM-CCSD formalisms. The results showed that electronic states with bound regions in the 20,000–40,000 cm^–1^ range were destabilized by the repulsive limbs of states arising from the Cu(3d^9^4s^2^, ^2^D_g_) + Be(2s^2^, ^1^S_g_) configuration. The theoretical calculations predicted a bound 2^2^Π state that is likely to be stable with respect to predissociation. However, observation of this state would be challenging due to the low oscillator strength of the 2^2^Π–X1^2^Σ^+^ transition. Owing to the nondiagonal distribution of Franck–Condon factors predicted for the 2^2^Π–X1^2^Σ^+^ transition, it does not appear to be suitable for direct laser cooling.

## Supporting Information Available

Table of observed band centers for ^63^CuBe and ^65^CuBe; potential energy curves calculated using the EE-EOM-CCSD formalism; pointwise potential energy curves for CuBe calculated using the TD-DFT formalism; pointwise potential energy curves for CuBe calculated using the EE-EOM-CCSD formalism (PDF)

## References

[ref1] Ladjimi H.; Tomza M. Diatomic molecules of alkali-metal and alkaline-earth-metal atoms: Interaction potentials, dipole moments, and polarizabilities. Phys. Rev. A 2024, 109 (5), 052814 10.1103/PhysRevA.109.052814.

[ref2] You Y.; Yang C.-L.; Wang M.-S.; Ma X.-G.; Liu W.-W. Theoretical investigation of the laser cooling of a LiBe molecule. Phys. Rev. A 2015, 92, 032502 10.1103/PhysRevA.92.032502.

[ref3] Pototschnig J. V.; Krois G.; Lackner F.; Ernst W. E. Investigation of the RbCa molecule: Experiment and theory. J. Mol. Spectrosc. 2015, 310, 126–134. 10.1016/j.jms.2015.01.006. 25922550 PMC4407902

[ref4] Persinger T. D.; Han J.; Heaven M. C. Electronic Spectroscopy and Photoionization of LiBe. J. Phys. Chem. A 2021, 125, 8274–8281. 10.1021/acs.jpca.1c07014. 34520195

[ref5] Persinger T. D.; Han J.; Heaven M. C. Electronic Spectroscopy and Photoionization of LiMg. J. Phys. Chem. A 2021, 125, 3653–3663. 10.1021/acs.jpca.1c01656. 33882672

[ref6] Pototschnig J. V.; Krois G.; Lackner F.; Ernst W. E. Ab initio study of the RbSr electronic structure: Potential energy curves, transition dipole moments, and permanent electric dipole moments. J. Chem. Phys. 2014, 141, 234309 10.1063/1.4903791. 25527937

[ref7] Lackner F.; Krois G.; Buchsteiner T.; Pototschnig J. V.; Ernst W. E. Helium-droplet-assisted preparation of cold RbSr molecules. Phys. Rev. Lett. 2014, 113, 153001 10.1103/PhysRevLett.113.153001. 25375707

[ref8] Gopakumar G.; Abe M.; Hada M.; Kajita M. Dipole polarizability of alkali-metal (Na, K, Rb)-alkaline-earth-metal (Ca, Sr) polar molecules: Prospects for alignment. J. Chem. Phys. 2014, 140, 224303 10.1063/1.4881396. 24929384

[ref9] Gao Y.; Gao T. Ab initio study of ground and low-lying excited states of MgLi and MgLi^+^ molecules with valence full configuration interaction and MRCI method. Mol. Phys. 2014, 112, 3015–3023. 10.1080/00268976.2014.926030.

[ref10] Stein A.; Ivanova M.; Pashov A.; Knoeckel H.; Tiemann E. Spectroscopic study of the 2^2^∑^+^ and the 4^2^∑^+^ excited states of LiCa. J. Chem. Phys. 2013, 138 (11), 114306 10.1063/1.4795205. 23534638

[ref11] Augustovičová L.; Soldan P. Ab initio properties of MgAlk (Alk = Li, Na, K, Rb, Cs). J. Chem. Phys. 2012, 136, 084311 10.1063/1.3690459. 22380046

[ref12] Gopakumar G.; Abe M.; Kajita M.; Hada M. Ab initio study of permanent electric dipole moment and radiative lifetimes of alkaline-earth-metal--Li molecules. Phys. Rev. A 2011, 84, 062514 10.1103/PhysRevA.84.062514.

[ref13] Ivanova M.; Stein A.; Pashov A.; Stolyarov A. V.; Knoeckel H.; Tiemann E. The X^2^∑^+^ state of LiCa studied by Fourier-transform spectroscopy. J. Chem. Phys. 2011, 135, 174303 10.1063/1.3652755. 22070298

[ref14] Kotochigova S.; Petrov A.; Linnik M.; Klos J.; Julienne P. S. Ab initio properties of Li-group-II molecules for ultracold matter studies. J. Chem. Phys. 2011, 135, 164108 10.1063/1.3653974. 22047229

[ref15] Russon L. M.; Rothschopf G. K.; Morse M. D.; Boldyrev A. I.; Simons J. Two-photon ionization spectroscopy and all-electron ab initio study of LiCa. J. Chem. Phys. 1998, 109, 6655–6665. 10.1063/1.477317.

[ref16] Śmiałkowski M.; Tomza M. Highly polar molecules consisting of a copper or silver atom interacting with an alkali-metal or alkaline-earth-metal atom. Phys. Rev. A 2021, 103, 022802 10.1103/PhysRevA.103.022802.

[ref17] Balducci G.; Ciccioli A.; Gigli G.; Kudin L. S. Mass spectrometric determination of the dissociation energy of the AuMg diatomic molecule. Chem. Phys. Lett. 2003, 369, 449–453. 10.1016/S0009-2614(02)02022-5.

[ref18] Balducci G.; Ciccioli A.; Gigli G. A mass spectrometric and density functional study of the intermetallic molecules AuBe, AuMg, and AuCa. J. Chem. Phys. 2004, 121, 7748–7755. 10.1063/1.1793971. 15485236

[ref19] Ciccioli A.; Gigli G.; Lauricella M. Experimental and computational investigation of the group 11-group 2 diatomic molecules: First determination of the AuSr and AuBa bond energies and thermodynamic stability of the copper- and silver-alkaline earth species. J. Chem. Phys. 2012, 136, 184306 10.1063/1.4711085. 22583286

[ref20] Barrow R. F.; Gissane W. J. M.; Travis D. N. Electronic spectra of some gaseous diatomic compounds of gold. Nature 1964, 201 (4919), 603–604. 10.1038/201603a0.

[ref21] Barrow R. F.; Gissane W. J. M.; Travis D. N. Rotational analysis of the A-X and B-X systems of AuBe and AuMg. Proc. R. Soc. London, Ser. A 1965, 287 (1409), 240–258. 10.1098/rspa.1965.0178.

[ref22] Coquant C.; Houdart R. Rotational structure analysis of near infrared bands of gold-calcium (AuCa) and gold-silicon (AuSi) compounds C. R. Hebd. Seances Acad. Sci., Ser. B 1977; Vol. 284, pp 171–172.

[ref23] Schiltz J. Spectrum of the AuCa molecule in the visible region Compt. Rend. 1961; Vol. 252, pp 1750–1752.

[ref24] Schlitz J. Optical spectra of gold compounds with alkaline earths Ann. Phys., 1963; Vol. 8 (1–2), , pp 62–106.

[ref25] Bauschlicher C. W. Jr.; Langhoff S. R.; Partridge H.; Walch S. P. Mixed copper-simple metal dimers and trimers: copper-lithium, -sodium, -potassium, -beryllium, and -aluminum (CuLi, CuLi_2_, CuNa, CuK, CuBe, CuBe_2_, Cu_2_Be, CuAl, and CuAl_2_). J. Chem. Phys. 1987, 86, 5603 10.1063/1.452536.

[ref26] NIST Atomic Spectra Database. http://www.nist.gov/pml/data/asd.cfm. (accessed January 2025).

[ref27] Western C. M. PGOPHER: A program for simulating rotational, vibrational and electronic spectra. J. Quant. Spectrosc. Radiat. Transfer 2017, 186, 221–242. 10.1016/j.jqsrt.2016.04.010.

[ref28] Bauschlicher C. W. Jr.; Langhoff S. R.; Partridge H. Theoretical study of the beryllium-lithium, beryllium-sodium, magnesium-lithium, magnesium-sodium, and aluminum-beryllium (BeLi, BeNa, MgLi, MgNa, and AlBe) molecules and their negative ions. J. Chem. Phys. 1992, 96, 1240–1247. 10.1063/1.462160.

[ref29] Smith D. G. A.; Burns L. A.; Simmonett A. C.; Parrish R. M.; Schieber M. C.; Galvelis R.; Kraus P.; Kruse H.; Di Remigio R.; Alenaizan A.; et al. PSI4 1.4: Open-source software for high-throughput quantum chemistry. J. Chem. Phys. 2020, 152 (18), 184108 10.1063/5.0006002. 32414239 PMC7228781

[ref30] Balabanov N. B.; Peterson K. A. Systematically convergent basis sets for transition metals. I. All-electron correlation consistent basis sets for the 3d elements Sc–Zn. J. Chem. Phys. 2005, 123, 064107 10.1063/1.1998907. 16122300

[ref31] Prascher B. P.; Woon D. E.; Peterson K. A.; Dunning T. H.; Wilson A. K. Gaussian basis sets for use in correlated molecular calculations. VII. Valence, core-valence, and scalar relativistic basis sets for Li, Be, Na, and Mg. Theor. Chem. Acc. 2011, 128 (1), 69–82. 10.1007/s00214-010-0764-0.

[ref32] Wolf A.; Reiher M.; Hess B. A. The generalized Douglas–Kroll transformation. J. Chem. Phys. 2002, 117 (20), 9215–9226. 10.1063/1.1515314. 15267790

[ref33] Neese F. Software update: the ORCA program system, version 4.0. WIREs Comput. Mol. Sci. 2018, 8 (1), e1327 10.1002/wcms.1327.

[ref34] Stanton J. F.; Bartlett R. J. The equation of motion coupled-cluster method. A systematic biorthogonal approach to molecular excitation energies, transition probabilities, and excited state properties. J. Chem. Phys. 1993, 98, 7029–7039. 10.1063/1.464746.

[ref35] van Vleck J. H. On L-Type Doubling and Electron Spin in the Spectra of Diatomic Molecules. Phys. Rev. 1929, 33, 467 10.1103/PhysRev.33.467.

[ref36] Mulliken R. S.; Christy A. L-Type Doubling and Electron Configurations in Diatomic Molecules. Phys. Rev. 1931, 38, 87 10.1103/PhysRev.38.87.

