# Supplementary material for: Electronic Structure and Spectroscopy of CuBe
Source: J Phys Chem A. 2025 Feb 13;129(8):2041–8. doi: 10.1021/acs.jpca.4c08565 (PMC11873972; doi:10.1021/acs.jpca.4c08565)
Supplement: Supplementary file 1 — jp4c08565_si_001.pdf [file jp4c08565_si_001.pdf]

Supporting Information for Publication

# Electronic Structure and Spectroscopy of CuBe

Arianna Rodriguez, Sophia Vadachkoria, Francesco A. Evangelista and Michael C. Heaven*

Arianna Rodriguez, Department of Chemistry, Emory University, Atlanta GA 30322, USA
Sophia Vadachkoria, Department of Chemistry, Emory University, Atlanta GA 30322, USA
Francesco A. Evangelista, Department of Chemistry, Emory University, Atlanta GA 30322, USA
Orcid ID 0000-0002-7917-6652
Michael C. Heaven*, Department of Chemistry, Emory University, Atlanta GA 30322, USA
Email: mheaven@emory.edu, Orcid ID 0000-0003-4738-2408

Table S1. Band positions and isotope shifts for CuBe

| $^{63}$CuBe cm$^{-1}$ | $^{65}$CuBe cm$^{-1}$ | Shift cm$^{-1}$ | Electronic state | v' |
|---|---|---|---|---|
| | | | | |
| 31018.2 | 31015.8 | 2.4 | $4^2\Sigma^+$ | 4 |
| 31373.1 | 31368.9 | 3.2 | $4^2\Sigma^+$ | 5 |
| 31715.4 | 31711.6 | 3.8 | $4^2\Sigma^+$ | 6 |
| 32058.4 | 32053.9 | 4.5 | $4^2\Sigma^+$ | 7 |
| 32284.5 | 32285.3 | -0.8 | | |
| 32391.5 | 32391.3 | 0.1 | $5^2\Sigma^+$ | 0 |
| 32509.7 | 32509.9 | -0.2 | | |
| 32712.3 | 32706.8 | 5.5 | $4^2\Sigma^+$ | 9 |
| 32739.8 | 32740.8 | -1.0 | | |
| 32776.8 | 32776.2 | 0.6 | $5^2\Sigma^+$ | 1 |
| 32871.1 | 32871.0 | 0.1 | $3^2\Pi_{1/2}$ | 0 |
| 32916.7 | 32916.0 | 0.7 | $3^2\Pi_{3/2}$ | 0 |
| 33167.6 | 33167.7 | -0.1 | $3^2\Pi_{1/2}$ | 1 |
| 33211.7 | 33211.9 | -0.2 | $3^2\Pi_{3/2}$ | 1 |
| 33431.0 | 33430.4 | 0.6 | $3^2\Pi_{1/2}$ | 2 |
| 33461.1 | 33461.9 | -0.8 | $3^2\Pi_{3/2}$ | 2 |
| 33586.3 | 33585.1 | 1.2 | $4^2\Pi_{1/2}$ | 0 |
| 33623.2 | 33622.7 | 0.5 | $4^2\Pi_{3/2}$ | 0 |
| 33690.6 | 33689.8 | 0.8 | $3^2\Pi_{1/2}$ | 3 |
| 33719.4 | 33719.9 | -0.5 | $3^2\Pi_{3/2}$ | 3 |
| 33865.3 | 33862.7 | 2.6 | | |
| 33937.1 | 33931.1 | 6.0 | | |
| 33997.7 | 33995.8 | 1.9 | $4^2\Pi_{1/2}$ | 1 |
| 34017.7 | 34017.4 | 0.3 | | |
| 34040.9 | 34039.8 | 1.1 | $4^2\Pi_{3/2}$ | 1 |
| 34075.5 | 34073.0 | 2.5 | | |
| 34107.4 | 34106.2 | 1.2 | | |
| 34334.9 | 34333.5 | 1.4 | | |
| 34406.7 | 34404.4 | 2.3 | $4^2\Pi_{1/2}$ | 2 |
| 34434.8 | 34432.0 | 2.8 | | |
| 34456.5 | 34454.8 | 1.6 | $4^2\Pi_{3/2}$ | 2 |
| 34597.4 | 34596.6 | 0.8 | | |
| 34618.8 | 34617.9 | 0.8 | | |

| | | | | |
|---|---|---|---|---|
| 34693.6 | 34690.9 | 2.7 | | |
| 34712.3 | 34710.4 | 1.9 | | |
| 34748.1 | 34748.3 | -0.1 | | |
| 34802.1 | 34799.3 | 2.8 | $4^2\Pi_{1/2}$ | 3 |
| 34829.0 | 34827.9 | 1.1 | | |
| 34843.5 | 34841.5 | 2.0 | $4^2\Pi_{3/2}$ | 3 |
| 35111.9 | 35111.8 | 0.1 | | |
| 35152.2 | 35148.1 | 4.1 | | |
| 35176.0 | 35174.4 | 1.6 | $4^2\Pi_{1/2}$ | 4 |
| 35202.3 | 35198.5 | 3.8 | | |
| 35211.9 | 35209.6 | 2.3 | $4^2\Pi_{3/2}$ | 4 |
| 35226.3 | 35222.5 | 3.7 | | |

1σ-errors, ±1

Fig. S1. Potential energy curves for CuBe calculated using the EE-EOM-CCSD formalism

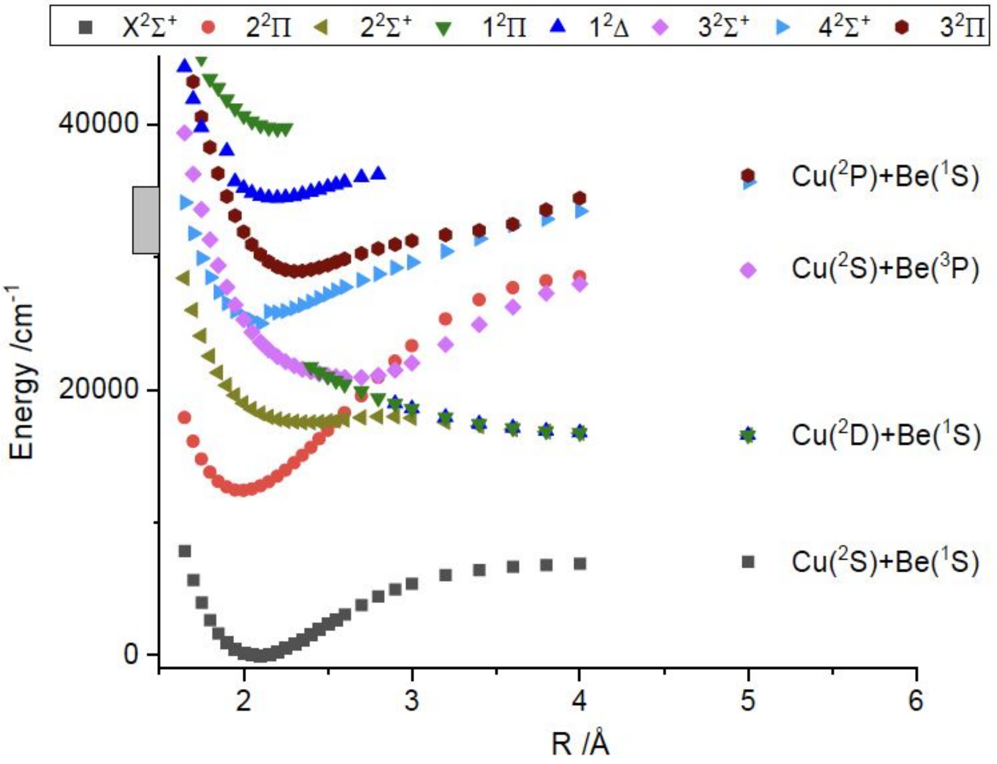


These single point energies were obtained using the Orca 6.0.1 software package. Scalar relativistic effects were included using the second-order approximation for the Douglas-Kroll-Hess Hamiltonian. The cc-pwCVTZ-DK basis sets were used for both Cu and Be. Stubborn discontinuities and root flipping problems were encountered in all attempts to apply the EE-EOM-CCSD method. Dramatic discontinuities are evident in the interatomic separation range from 2.2 to 3.0 A. Despite these problems, it is still clear that the higher excited states are likely to be predissociated by the repulsive limbs of the potential energy curves derived from the $Cu(3d^9 4s^2, {}^2D)+Be(2s^2, {}^1S)$ atomic limit.

Table S2. Pointwise potential energy curves for CuBe calculated using the TD-DFT formalism

| | $X1^2\Sigma^+$ | $2^2\Pi$ | $2^2\Pi$ | $1^2\Delta$ | $1^2\Delta$ | $1^2\Pi$ | $3^2\Sigma^+$ | $1^2\Pi$ | $2^2\Sigma^+$ | $4^2\Sigma^+$ | $3^2\Pi$ |
|---|---|---|---|---|---|---|---|---|---|---|---|
| R | | | | | | | | | | | |
| 1.7 | 5149.7 | 16831.7 | 16831.7 | 39843.1 | 39771.3 | 33632.6 | 36180.0 | 33632.6 | 27064.0 | 40698.5 | 41513.0 |
| 1.8 | 2232.0 | 14868.1 | 14868.1 | 33856.6 | 33792.0 | 30298.6 | 31652.1 | 30298.6 | 23678.5 | 36781.9 | 37136.7 |
| 1.9 | 642.4 | 14111.7 | 14111.7 | 29535.2 | 29476.8 | 28279.5 | 28497.4 | 28279.5 | 21600.5 | 31932.5 | 34368.7 |
| 2 | 0.0 | 14184.6 | 14184.6 | 27116.1 | 27116.1 | 26418.7 | 26454.4 | 26401.1 | 20375.5 | 28525.6 | 32790.4 |
| 2.1 | 13.7 | 14804.6 | 14804.6 | 25560.6 | 25560.6 | 24286.5 | 25152.1 | 24236.7 | 19668.9 | 27203.9 | 32080.4 |
| 2.2 | 452.6 | 15757.2 | 15757.2 | 23840.9 | 23840.9 | 22765.3 | 24451.3 | 22719.6 | 19255.2 | 27233.7 | 31980.9 |
| 2.3 | 1179.5 | 16923.8 | 16923.8 | 22595.3 | 22595.3 | 21730.6 | 24138.3 | 21689.3 | 19044.6 | 27654.1 | 30825.7 |
| 2.4 | 2067.9 | 18198.7 | 18198.7 | 21727.3 | 21727.3 | 21039.5 | 24053.7 | 21001.8 | 18967.0 | 28264.3 | 30723.8 |
| 2.5 | 3029.6 | 19514.2 | 19514.2 | 21133.4 | 20553.2 | 21133.4 | 24105.4 | 20587.8 | 18981.5 | 28957.5 | 30942.2 |
| 2.6 | 4012.8 | 20840.1 | 20840.1 | 20311.4 | 20738.1 | 20738.1 | 24260.6 | 20280.2 | 19068.9 | 29674.0 | 31393.1 |
| 2.7 | 4968.2 | 22122.3 | 22122.3 | 20152.7 | 20492.8 | 20492.8 | 24490.6 | 20124.7 | 19195.2 | 30365.3 | 31981.0 |
| 2.8 | 5860.1 | 23342.5 | 23342.5 | 20067.7 | 20334.5 | 20334.5 | 24769.3 | 20042.1 | 19330.8 | 31008.4 | 32623.7 |
| 2.9 | 6675.1 | 24478.8 | 24478.8 | 20033.3 | 20241.7 | 20241.7 | 25081.4 | 20010.0 | 19467.1 | 31610.6 | 33266.1 |
| 3 | 7403.6 | 25401.1 | 25510.1 | 20031.3 | 20192.6 | 20192.6 | 25510.1 | 20010.3 | 19596.7 | 32183.1 | 33865.7 |
| 3.1 | 8037.6 | 26417.5 | 26417.5 | 20044.7 | 20168.1 | 20168.1 | 25707.3 | 19710.4 | 20025.9 | 32734.0 | 34393.8 |
| 3.2 | 8579.7 | 27193.7 | 27193.7 | 20065.8 | 20158.5 | 20158.5 | 25986.6 | 19807.4 | 20048.8 | 33274.3 | 34846.9 |
| 3.4 | 9424.5 | 28378.2 | 28378.2 | 20122.9 | 20171.4 | 20171.4 | 26460.4 | 19965.3 | 20108.3 | 34343.9 | 35575.9 |
| 3.6 | 9999.8 | 29156.3 | 29156.3 | 20176.6 | 20199.6 | 20199.6 | 26844.4 | 20076.0 | 20163.8 | 35355.1 | 35368.7 |
| 3.8 | 10383.1 | 29664.8 | 29664.8 | 20224.6 | 20233.1 | 20233.1 | 27191.4 | 20152.8 | 20212.9 | 36257.4 | 36271.0 |
| 4 | 10631.9 | 29996.1 | 29996.1 | 20259.5 | 20259.5 | 20261.2 | 27523.3 | 20216.7 | 20250.4 | 37019.6 | 37033.3 |
| 4.2 | 10791.9 | 30219.1 | 30219.1 | 20282.4 | 20282.4 | 20289.5 | 27857.5 | 20256.9 | 20279.3 | 37649.0 | 37662.7 |
| 4.4 | 10895.5 | 30369.0 | 30369.0 | 20302.5 | 20302.5 | 20307.7 | 28176.0 | 20282.0 | 20297.7 | 37693.6 | 38179.4 |
| 4.6 | 10959.2 | 30464.7 | 30464.7 | 20312.7 | 20312.7 | 20318.4 | 28477.1 | 20302.5 | 20308.9 | 37981.8 | 38593.4 |
| 5 | 11025.4 | 30567.3 | 30567.3 | 20320.7 | 20328.5 | 20329.1 | 29039.3 | 20319.9 | 20319.9 | 38493.4 | 39221.7 |

Table S3. Pointwise potential energy curves for CuBe calculated using the EE-EOM-CCSD formalism

| | $X1^2\Sigma^+$ | $2^2\Pi$ | $2^2\Pi$ | $2^2\Sigma^+$ | $4^2\Sigma^+$ | $3^2\Pi$ | $1^2\Pi$ | $1^2\Delta$ | $3^2\Sigma^+$ | $1^2\Pi$ |
|---|---|---|---|---|---|---|---|---|---|---|
| R | | | | | | | | | | |
| 1.65 | 7862.4 | 17898.8 | 17898.8 | 28431.6 | 34112.4 | 34112.4 | 46411.1 | 44335.5 | 39382.9 | 48739.3 |
| 1.7 | 5693.7 | 16122.7 | 16122.7 | 26021.7 | 31787.3 | 31787.3 | 43225.2 | 41918.6 | 36262.6 | 46718.5 |
| 1.75 | 3981.1 | 14774.0 | 14774.0 | 24081.5 | 29931.3 | 29931.3 | 40557.4 | 39794.5 | 33590.9 | 45055.1 |
| 1.8 | 2655.4 | 13785.5 | 13785.5 | 22530.4 | 28474.0 | 28474.0 | 38283.2 | 53353.8 | 31313.5 | 43471.3 |
| 1.85 | 1657.0 | 13100.9 | 13100.9 | 21300.6 | 27361.4 | 27361.4 | 36308.1 | 51831.9 | 29383.7 | 42799.0 |
| 1.9 | 934.2 | 12669.8 | 12669.8 | 20332.5 | 26534.2 | 26534.2 | 34591.0 | 37999.7 | 27757.3 | 41926.7 |
| 1.95 | 442.8 | 12450.7 | 12450.7 | 19577.0 | 25943.8 | 25943.8 | 33134.2 | 35696.7 | 26400.5 | 41245.3 |
| 2 | 144.3 | 12408.8 | 12408.8 | 18992.4 | 25541.3 | 25541.3 | 31928.0 | 35202.5 | 25274.8 | 40670.7 |
| 2.05 | 6.3 | 12513.5 | 12513.5 | 18545.3 | 25263.0 | 25264.3 | 30960.1 | 34831.4 | 24351.5 | 40253.9 |
| 2.1 | 0.0 | 12740.6 | 12740.6 | 18207.6 | 24994.9 | 24994.9 | 30210.5 | 34620.6 | 23601.0 | 39959.9 |
| 2.15 | 100.7 | 13067.6 | 13067.6 | 17957.9 | 25895.8 | 25895.8 | 29657.2 | 34492.2 | 22996.1 | 39784.1 |
| 2.2 | 287.6 | 13477.5 | 13477.5 | 17778.9 | 25827.0 | 25827.0 | 29276.5 | 34459.0 | 22512.3 | 39723.5 |
| 2.25 | 541.6 | 13953.9 | 13953.9 | 17657.1 | 25940.3 | 25940.3 | 29048.0 | 34503.3 | 22125.6 | 39766.3 |
| 2.3 | 846.8 | 14484.3 | 14484.3 | 17582.7 | 26133.8 | 26133.8 | 28950.5 | 34595.6 | 21817.4 | 45994.7 |
| 2.35 | 1189.3 | 15057.9 | 15057.9 | 17547.4 | 26373.8 | 26373.8 | 28960.7 | 34740.8 | 21570.6 | 46263.4 |
| 2.4 | 1556.8 | 15664.7 | 15664.7 | 17545.8 | 26639.7 | 26639.7 | 29061.4 | 34911.2 | 21374.7 | 21727.2 |
| 2.45 | 1939.5 | 16296.3 | 16296.3 | 17571.9 | 26918.6 | 26918.6 | 29219.3 | 35087.0 | 21217.3 | 21344.8 |
| 2.5 | 2327.5 | 16944.3 | 16944.3 | 17619.4 | 27199.5 | 27199.5 | 29416.6 | 35272.6 | 21091.9 | 21002.0 |
| 2.55 | 2713.6 | 17603.6 | 17603.6 | 17683.5 | 27478.0 | 27478.0 | 29638.9 | 35457.2 | 21001.3 | 20690.8 |
| 2.6 | 3091.5 | 18268.9 | 18268.9 | 17758.6 | 27751.1 | 27751.1 | 29864.2 | 35643.6 | 20940.1 | 20405.5 |
| 2.7 | 3802.7 | 19549.2 | 19549.2 | 17903.6 | 28268.0 | 28268.0 | 30286.5 | 36002.1 | 20930.8 | 19931.8 |
| 2.8 | 4433.0 | 20928.1 | 20928.1 | 17992.2 | 28742.6 | 28743.1 | 30655.8 | 36212.4 | 21103.0 | 19386.7 |
| 2.9 | 4969.5 | 22165.8 | 22165.8 | 17982.2 | 29186.6 | 29186.6 | 30969.3 | 18962.6 | 21476.0 | 18975.5 |
| 3 | 5411.6 | 23327.7 | 23327.7 | 17886.5 | 29605.6 | 29605.6 | 31236.1 | 18573.9 | 22023.0 | 18584.3 |
| 3.2 | 6047.4 | 25329.4 | 25329.4 | 17576.4 | 30444.9 | 30444.9 | 31674.1 | 17926.5 | 23419.3 | 17931.3 |
| 3.4 | 6435.4 | 26782.2 | 26782.2 | 17264.5 | 31404.6 | 31404.6 | 32030.5 | 17451.1 | 24914.5 | 17449.1 |
| 3.6 | 6666.9 | 27692.3 | 27692.3 | 17022.6 | 32405.1 | 32493.3 | 32493.3 | 17121.6 | 26249.5 | 17119.0 |
| 3.8 | 6805.5 | 28219.9 | 28219.9 | 16852.4 | 32882.4 | 33562.5 | 33562.5 | 16905.8 | 27285.1 | 16901.6 |
| 4 | 6892.8 | 28529.3 | 28529.3 | 16742.0 | 33463.8 | 34465.0 | 34465.0 | 16771.0 | 27991.4 | 16766.2 |
| 5 | 7055.4 | 29019.8 | 29019.8 | 16591.6 | 35662.8 | 36173.1 | 36169.1 | 16589.6 | 29007.0 | 16592.5 |
